# GenoPath: a pipeline to infer tumor clone composition, mutational history, and metastatic cell migration events from tumor DNA sequencing data

**DOI:** 10.3389/fbinf.2025.1615834

**Published:** 2025-07-02

**Authors:** Ryan M. Tobin, Shikha Singh, Sudhir Kumar, Sayaka Miura

**Affiliations:** ^1^ Department of Biology, Temple University, Philadelphia, PA, United States; ^2^ Institute of Genomic and Evolutionary Medicine, Temple University, Philadelphia, PA, United States; ^3^ Department of Biology, University of Mississippi, University, MS, United States

**Keywords:** evolution, cancer, sequencing, somatic mutation, tumor, phylogency, mutational process, metastasis

## Abstract

DNA sequencing technologies are widely used to study tumor evolution within a cancer patient. However, analyses require various computational methods, including those to infer clone sequences (genotypes of cancer cell populations), clone frequencies within each tumor sample, clone phylogeny, mutational tree, dynamics of mutational signatures, and metastatic cell migration events. Therefore, we developed GenoPath, a streamlined pipeline of existing tools to perform tumor evolution analysis. We also developed and added tools to visualize results to assist interpretation and derive biological insights. We have illustrated GenoPath’s utility through a case study of tumor evolution using metastatic prostate cancer data. By reducing computational barriers, GenoPath broadens access to tumor evolution analysis. The software is available at https://github.com/SayakaMiura/GP.

## Introduction

Cancer progression is driven by an accumulation of somatic mutations (Pleasance et al., 2010; Alexandrov and Stratton, 2014; Martincorena and Campbell, 2015; Martincorena, 2019). These mutations make tumors a genetically diverse population of cancer cells (Marusyk et al., 2012; 2020; Cajal et al., 2020; Vitale et al., 2021). Bulk DNA sequencing and data analysis are widely used to elucidate patterns of tumor evolution, including the timing of driver mutations, shifts in mutational processes, and inferences of metastatic migration paths (Jamal-Hanjani et al., 2017; Turajlic et al., 2018; Al Bakir et al., 2023; Frankell et al., 2023).

To investigate the characteristics of tumor evolution, reconstruction of the phylogeny of tumor cell populations (clone phylogeny) and analysis of the inferred phylogeny are essential (Schwartz and Schäffer, 2017; Stadler et al., 2021). Each branch of a clone phylogeny represents the accumulation of mutations, and branching events reflect the genetic diversification of cancer cell populations (tumor clones). By mapping mutations onto a clone phylogeny, researchers can reconstruct mutational history and elucidate the timing of driver mutation occurrences. Temporal dynamics of mutational processes can be investigated by identifying branch-specific mutational signatures. Additionally, the metastatic cell migration history can be reconstructed when both primary and metastatic tumor samples are sequenced.

However, practical tumor evolution analyses can be challenging. Since bulk sequencing captures the aggregate genetic composition of heterogeneous cell populations rather than individual cells, such mutation profiles must first be deconvoluted into distinct clone sequences. This requires specialized bioinformatics tools (Miura et al., 2020; Laganà, 2022). Also, downstream analyses (e.g., clone phylogeny and mutational tree reconstruction, mutational signature analysis, and metastatic migration inference) need additional computational methods and tools (El-Kebir et al., 2018; Christensen et al., 2020; Kumar et al., 2020; Miura et al., 2022). Furthermore, interpreting output files is often difficult due to the lack of a universal data format in tumor evolution analysis. These factors create a barrier for researchers without computational expertise.

We developed GenoPath, a Python-based command-line tool that streamlines tumor evolution analysis to address these challenges. GenoPath processes variant read counts (bulk DNA sequencing data) from each tumor sample from the same patient and executes various tumor evolution analyses. It infers clone sequences, clone phylogenies, and mutational trees; identifies shifts of mutational signatures and timing of driver mutation occurrences during tumor evolution; and reconstructs metastatic cell migration events. We also developed tools to visualize these results. GenoPath makes tumor evolution analysis accessible to researchers with varying expertise levels.

## Methods

### Overview of GenoPath

In the GenoPath pipeline, we integrated existing software that had been previously benchmarked in the original studies, i.e., CloneFinder (Miura et al., 2018), PhyloSignare (Miura et al., 2022), PathFinder (Kumar et al., 2020), MEGA (Kumar et al., 2012), and picante (Kembel et al., 2010). Since each tool requires a specific input format, we developed preprocessing modules to parse and format input files within the pipeline. Additionally, many of these computational methods lack intuitive visual outputs, so we also developed visualization tools to enhance the clarity of evolutionary inferences. GenoPath is written in Python.

**FIGURE 1:**
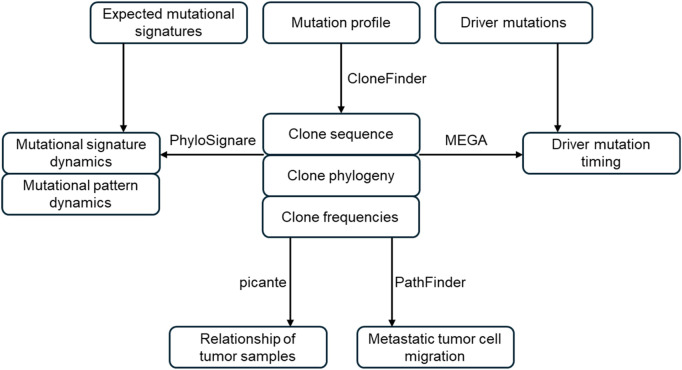
Overview of GenoPath pipeline. GenoPath infers clone sequences, clone frequencies, and clone phylogeny using the observed read counts. The inferred clone phylogeny and sequences are used to map driver mutations to branches and analyze the evolutionary dynamics of mutational signatures. Also, clone phylogeny and clone frequencies are utilized to reconstruct metastatic tumor cell migration history and build a tumor sample tree. GenoPath produces intuitive visualizations for each analysis, aiding in the interpretation and analysis of tumor evolution.

For GenoPath analysis, users are required to provide reference (non-mutant) and mutant read counts for each variant position across tumor samples (mutation profile), along with a list of driver mutations and expected mutational signatures (Figure 1). In a given mutation profile, the information on non-mutant base, mutant base, and trinucleotide are additionally required for each variant. GenoPath first infers clone sequences, clone frequencies, and clone phylogeny from the mutation profile. GenoPath utilizes CloneFinder for this step. Due to the requirement in CloneFinder, at least four tumor samples from the same patient are necessary. CloneFinder infers clone sequences (*M*) and their frequencies (*f*) from observed variant frequencies (*V*) by solving *f* × *M* = *V*, under the assumption that variant are not affected by copy number alterations (CNAs). It further models tumor samples as being evolutionarily related and searches for *M* and *f*. Using inferred clone sequences, CloneFinder produces a maximum parsimony phylogeny, which represents the evolutionary relationships among distinct tumor clones in a patient. Each tip corresponds to a clone. The branching patterns denote their ancestral relationships, which reflect how clones have diverged from one another over time. We developed a visualization tool so GenoPath can produce an intuitive graphic of inferred clone phylogeny and clone frequencies, which was not included in CloneFinder.

GenoPath next builds a mutational tree (temporal order of somatic mutations) using the inferred clone sequences and clone phylogeny. In a mutation tree, nodes correspond to groups of mutations that occurred concurrently, and edges capture their inferred order of accumulation during tumor evolution. To infer a mutational tree, ancestral sequences are reconstructed at internal nodes using the Maximum Parsimony approach implemented in MEGA-CC (Kumar et al., 2012), which is appropriate because the sequence divergence is relatively low. A mutation is assigned to a branch when its base assignment differs from that of its direct ancestral clone. GenoPath accordingly maps driver mutations to branches of the clone phylogeny using a user-provided list of driver mutations. Lastly, GenoPath produces a graphic of the mutational tree with all driver mutations mapped to assist mutational history analysis.

A mutational tree is also used for the PhyloSignare analysis in the GenoPath pipeline. PhyloSignare identifies mutational signatures at each edge of a given mutational tree, i.e., branches of an inferred clone phylogeny. It uses an existing signature refitting method (default: quadratic programming) to estimate relative activities of signatures, which is followed by filtering out the spurious ones. The approach assumes that neighboring branches in the phylogeny share similar mutational signatures and that gains and losses of signatures are rare during tumor evolution. GenoPath enhances visualization for analyzing PhyloSignare results by generating a layered representation of the tumor’s mutational landscape, a feature not available in PhyloSignare. This visualization includes displays of the fraction of detected mutational signatures at each edge of the mutational tree (branch of clone phylogeny) and mutational profiles for each edge.

In addition, GenoPath reconstructs metastatic tumor cell migration history using inferred clone phylogeny and clone frequencies using PathFinder. PathFinder estimates Bayesian posterior probabilities of anatomical locations of ancestral clones based on a given evolutionary relationship of clones (clone phylogeny) and their location (clone frequency within tumor samples). Clone migration events are inferred when connected nodes in the phylogeny have different anatomical locations. Inferred clone migration events are summarized into a tumor cell migration graph. In a tumor cell migration graph, nodes represent physical tumor locations and directed edges indicate the inferred direction of clone movement.

GenoPath also builds a sample tree, which represents the similarity in clone composition between tumor samples. Using UniFrac and nearest-distance metrics in the Picante software, GenoPath calculates pairwise distances between tumor samples based on the similarity of their clone compositions. From the pairwise distance matrix, GenoPath builds a neighbor-joining tree (Saitou and Nei, 1987), which is the phylogeny of samples. A sample tree provides a sample-level view of tumor evolution, in contrast to the clone-level resolution of the clone phylogeny. Tumor samples that cluster closely in this phylogeny likely exchange clones, suggesting tumor cell migration events.

GenoPath applies default or recommended settings for each integrated software, which were determined in the original studies. It also outputs all parameter settings and options used, enabling reproducibility and transparency.

### Empirical data analysis

We obtained tumor DNA sequencing data (Patient GP12) from the Supplementary material of a previous study (Nurminen et al., 2023). The data input consists of the read counts of mutant and non-mutant alleles at each genomic position of single-nucleotide variants (SNVs) for each tumor sample. SNVs identified in at least one tumor sample from this patient were included. In total, 17,805 SNVs were included in this data. We also obtained trinucleotide information for each SNV from the supplementary material for the mutational signature analysis in GenoPath. Expected signatures (SBS1, SBS5, SBS8, SBS18, SBS3, SBS2, SBS13, SBS92, and SBS17) for prostate cancer were obtained from the Signal database (5 April 2025) (https://signal.mutationalsignatures.com/explore). Rare mutational signatures were not included. The list of driver mutations in the coding regions was also obtained from the supplementary material of the previous paper. We considered them driver mutations when they were found in the COSMIC database.

## Results

To illustrate the usage of GenoPath, we analyzed the prostate cancer dataset. This data was generated by performing whole-genome sequencing on eleven tumor samples from a single patient in a previous study (Nurminen et al., 2023). The primary tumor (CA) was divided into distinct subsections. These subsections included the base of the seminal vesicle (SVBase) and prostate. The prostate sample was further divided into apex (Apex) and middle (Mid) sites, where the Mid was then subdivided into basal (MidBasal) and apical (MidApical) sections. Each sample’s right (R) and left (L) sides were sequenced, resulting in eight primary tumor samples. In addition, three metastatic tumor samples were extracted from the right (R) and left (L) sides of the pelvic lymph nodes (PelvicLNMet).

### Evolutionary history of clones

**FIGURE 2:**
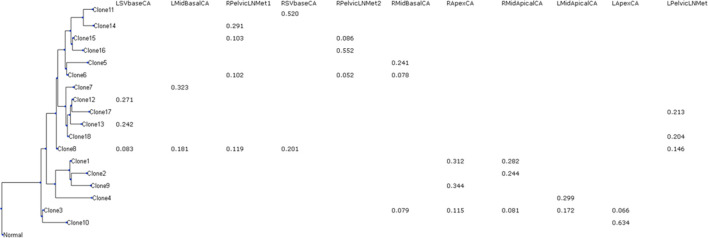
The display of clone phylogeny and clone frequencies generated by GenoPath. A tip of the phylogeny is an inferred clone. Clone frequencies are shown for each tumor sample. Zero clone frequencies are not shown. L/R, left/right; Mid, middle of the tumor; SV, seminal vesicle; LN, lymph node; CA, primary tumor; Met, metastasis.

The inferred clone phylogeny and clone frequencies produced by GenoPath are shown in Figure 2. Using this display, we can visually investigate the evolutionary history of clones and the distribution of clones among different tumor samples. The clone phylogeny is presented on the left side, where each tip of the phylogeny represents the observed (inferred) clone sequence. The branch lengths in the clone phylogeny correlate with the number of mutations that occurred along a branch. Longer branches indicate larger numbers of mutations. A branching event corresponds to the diversification of clones. GenoPath presents the estimated clone frequency within each tumor sample next to each tip of the phylogeny. Thus, the clone frequency table is aligned with the clone phylogeny. Often, clones are locally spread, and many tumor samples do not have a given clone. As a result, the clone frequency table may contain many zero values, which suggests an absence of those clones within a tumor sample. To easily view the pattern of the presence and absence of clones within tumor samples, GenoPath shows only non-zero values in the clone frequency table.

The CloneFinder analysis implemented in GenoPath produced 18 tumor clones for the example empirical dataset. GenoPath’s display of the inferred clone phylogeny and clone frequencies on this dataset is shown in Figure 2. We found that this display was helpful to visually investigate the distribution of clones among tumor samples and their evolutionary history. For example, Clone11 was found only within the right side of SVbase (RSVbaseCA). Since the left side of the SVbase, as well as the other sections of the primary tumor and metastatic tumor, did not have Clone11, this pattern of clone distribution suggested local expansion of the cancer cell population (Clone11). This RSVbaseCA tumor sample also contained another clone, Clone8, with a lower frequency than Clone11 (20% and 52%, respectively). By examining the display of the clone phylogeny, we found that Clone8 was an ancestral clone of Clone11. Our investigation of the clone frequency table revealed that Clone8 was widely present at the left side of the primary tumor, except at the apex (LApexCA) and middle-apical site (LMidApicalCA). Also, Clone8 was detected at the right side of the seminal vesicle (RSVbaseCA) and the right side of the metastatic lymph node (RPelvicLNMet1). This pattern suggested that the ancestral clone, Clone8, was widely spread, and additional mutations on Clone8 gave rise to Clone11. Then, Clone11 expanded only to a limited location within a tumor.

Available clone prediction methods do not produce such an intuitive display. Thus, we expect that using GenoPath would make it more accessible to analyze clone evolution and characterize intratumor heterogeneity for a given patient.

### Mutational tree and timing of driver mutations

Distinguishing early (truncal) from late (branched) driver mutations provides key insights into tumor evolution. Early drivers, shared across all tumor cells, are often essential for initiation. Late drivers contribute to progression, heterogeneity, and therapy resistance. Mapping these events onto the clone phylogeny (reconstruction of mutational tree) allows us to trace the tumor’s mutational history and understand lineage-specific dynamics. To assist in analyzing mutational history, we developed and implemented a tool to convert an inferred clone phylogeny to a mutational tree (see Methods). We also created a tool to visualize a mutational tree with user-provided driver mutations mapped.

**FIGURE 3:**
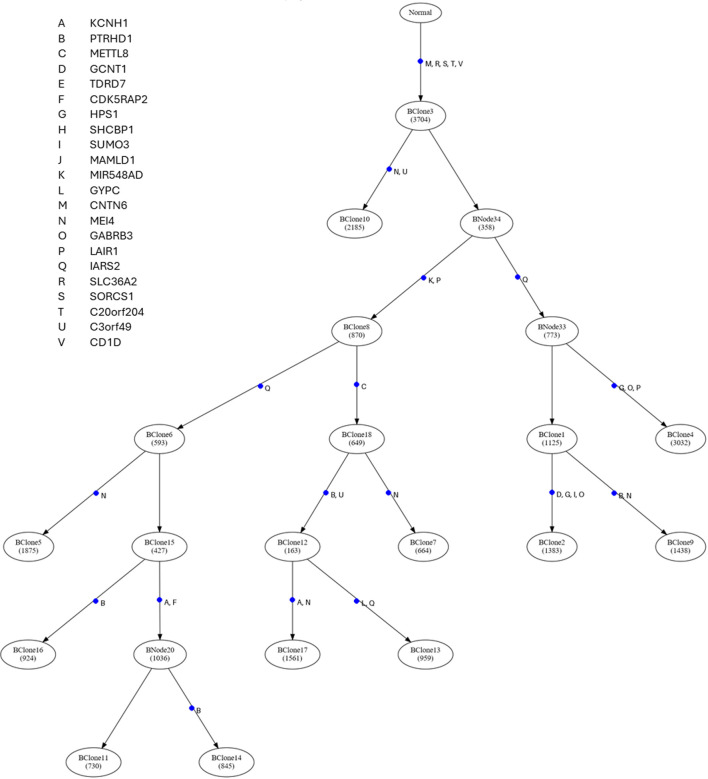
The display of the mutational tree and the timing of driver mutations generated by GenoPath. The number of mutations occurring along each edge of the mutational tree is indicated within a circle connected to an edge. Within each circle, the ID of the group of mutations that occurred at the same edge of the mutational tree is given. The timing of each driver mutation occurrence is indicated with a blue dot along the edge of the mutational tree. A driver mutation ID is listed next to each blue dot, and gene names are found on the top left side.

Figure 3 presents GenoPath’s visual of the mutational tree with driver mutations. In GenoPath’s display, the timing of driver mutation is highlighted with a blue point, and the names of given driver mutations are listed next to the blue point. In this way, users can easily investigate the timing of driver mutations during clone evolution. For example, in the analysis of our example dataset, driver mutations were mapped along different edges in addition to the trunk of the mutational tree (Figure 3). This result indicated that driver mutations occurred continuously during the evolution of clones. Also, driver mutations were mapped along different clonal lineages, suggesting that clones from different lineages have different sets of driver mutations. This result was consistent with a previous study, which reported that driver mutations may occur at both early and late times during tumor evolution (Gomez et al., 2018). Overall, with GenoPath, users can visually investigate the pattern of driver mutation occurrences during tumor evolution.

### Dynamics of mutational processes

**FIGURE 4:**
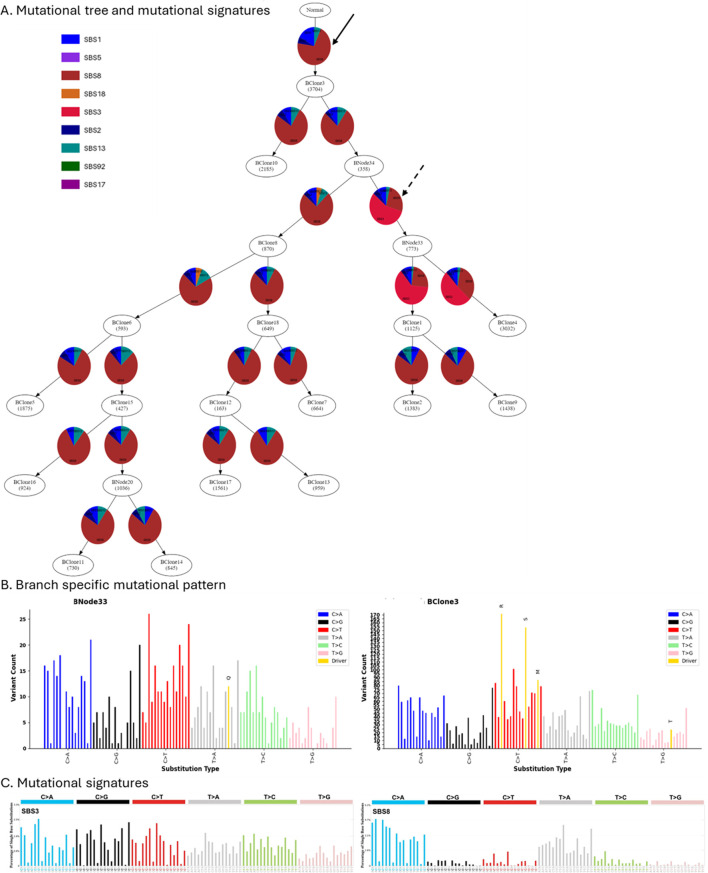
The display of the branch-specific mutational signatures and mutational patterns generated by GenoPath. **(A)** Mutation tree with mutational signatures. Inferred relative fractions of mutational signatures are shown along each edge of the mutation tree. **(B)** Observed mutation patterns at the edges of the mutation tree. Due to the space limitation, the mutational patterns of only two edges are shown. The left and right panels are the observed mutational patterns at the edges of the mutational tree that are pointed by dashed and solid arrows, respectively, in **(A) (C)** Mutational signatures SBS3 and SBS8. The plots were obtained from the COSMIC database.

When mutational processes alter during tumor evolution, observed mutational patterns change. As a result, different clonal lineages may have distinct mutational signatures. Next, we demonstrate how GenoPath can assist in analyzing the dynamics of mutational processes. Figure 4 presents displays that GenoPath produced. The estimated fractions of mutational signatures by PhyloSignare are shown on the edges of the mutational tree so that users can visually investigate the pattern of signature changes during the clone evolution (Figure 4A).

Our case study shows that all edges of the mutational tree had a high activity of mutational signature SBS8, with a few exceptions. SBS8 is characterized by elevated rates of specific C to A trinucleotide mutation types (Figure 4C). GenoPath assists users in visually comparing the characteristics of detected mutational signatures and observed mutational pattern by generating a plot of observed mutational pattern for each edge of the mutational tree (Figure 4B). In this case, the observed mutational patterns also had higher counts of the specific C to A trinucleotide mutation types. Consequently, observed mutational patterns agreed well with the characteristics of the detected signature (SBS8), validating the inference.

We also found that a few edges of the mutational tree had mutational signature SBS3 with a high fraction instead of SBS8 (Figure 4A). These results indicated a decrease and an increase of signature SBS8 and a gain and a loss of SBS3 activity. Interestingly, SBS3 also had the same characteristic as SBS8, where the same C to A trinucleotide mutation types had elevated rates (Figure 4C). Using the branch-specific mutational pattern produced by GenoPath, we confirmed that the observed mutational pattern also had higher counts of these same trinucleotide types (Figure 4B). Therefore, we cannot reliably distinguish SBS3 from SBS8, and the inferred decrease and increase of signature SBS8 and gain and loss of SBS3 activity were spurious. Overall, GenoPath’s visuals of observed mutational patterns, together with the inferred mutational signatures, are useful to validate the inferences, enabling more robust visual analysis.

We also designed GenoPath to assist in investigating the impacts of elevated mutation rates of mutation types on the occurrence of driver mutations, i.e., GenoPath highlights the mutation type of each driver mutation in the observed mutational pattern plot (Figure 4B). In this case study, GenoPath’s display showed that some driver mutations indeed occurred at mutational types with elevated mutation rates, e.g., mutations “R” and “S.” However, many other driver mutations did not follow this pattern, indicating that the impacts of elevated mutation rates of mutation types were variable among the driver mutations for this patient. Therefore, GenoPath can enable more comprehensive mutational analysis.

### Metastatic cell migration events and the relationship of tumor samples

**FIGURE 5:**
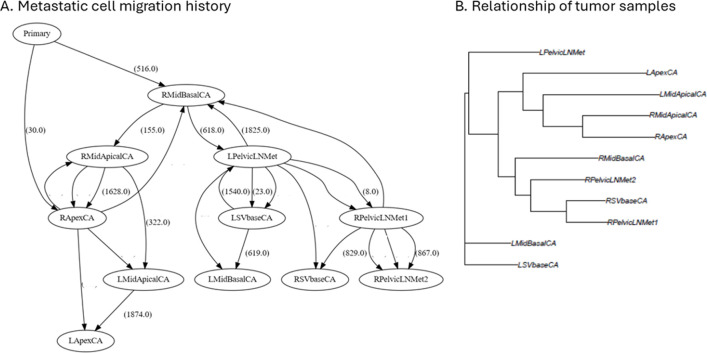
The display of metastatic cell migration history and tumor sample trees produced by GenoPath. **(A)** Metastatic cell migration history. The number in a parenthesis along an edge of the graph is the number of mutations associated with the cell migration event. Zero mutation is not shown. ID in a circle is a tumor sample. **(B)** Sample tree based on the similarity of clone composition. Tips of a sample tree are tumor samples. A sample tree indicates the relationship of tumor samples. L/R left/right; Mid, middle of the tumor; SV, seminal vesicle; LN, lymph node; CA, primary tumor; Met, metastasis.

During cancer progression, tumor cells may migrate to another tumor site, i.e., metastasis. With tumor sequencing data, metastatic cell migration history can be reconstructed. Figure 5A is the metastatic cell migration history inferred by PathFinder, which was implemented in GenoPath. In the inferred metastatic cell migration graph, each node is a tumor site, and tumor sites directly connected from the node of “Primary” are the sites of the tumor origin. Since the right side of the middle-apical and apex sites (RMidBaseCA and RapexCA) were directly connected from the “Primary” node, this area was predicted as the tumor initiation site. Overall, each tumor site was connected to and from various tumor sites, indicating a complex metastatic cell migration history.

In this inference, we found that the number of mutations associated with migration events was often zero (Figure 5A). Inference of migration events without associated mutations is known to be difficult to derive because the information to determine the direction of the migration event is limited. Therefore, careful inspection is essential to avoid implying spurious biological insight. One way to inspect uncertainty is by examining the intermediate inferences produced by PathFinder, where ambiguous migration paths may not appear consistently across all inferences. Although these intermediate results are not part of GenoPath’s main output, they are accessible for advanced users who wish to explore them. Also, GenoPath assists this validation process through the display of clone phylogeny together with clone frequencies, which is useful to identify potential clones that migrate to another tumor site without additional mutations, i.e., shared clones between tumor samples (Figure 2). The visual inspection found that clone sharing between tumor samples was extensive for this patient. For example, the earliest clone (Clone3) was found within five different tumor samples, making it difficult to infer the tumor initiation site confidently. Therefore, accurately determining metastatic cell migration events in this patient is challenging, and additional information is necessary to validate the inferred cell migration history.

GenoPath also provides an approach to analyzing the relationship of tumor samples. We implemented a tool to infer a tree of tumor samples based on the similarity of the composition of clones (see Methods). Tumor samples clustered together on the tree imply that these samples are closely related due to the spread or migration of cancer cells. Figure 5B shows the tumor sample tree of this case study. For example, two samples from the right side of the primary tumor (RMidBasalCA and RSVbaseCA) and two metastatic tumor samples from the right side of the lymph node (RPelvicLNMet1 and RPelvidLNMet2) were clustered in the tumor tree. This result suggested that cancer cells with similar genomic compositions were widely spread on the primary tumor’s right side and the lymph node’s right side. Similarly, the sample tree implied that the spread of other evolutionarily closely related cancer cells at the left side of the primary tumor (LmidBasalCA, LSVbaseCA) and the left side of the metastatic tumor (LPelvidLNMet), because these samples clustered together on the sample tree. Lastly, the sample tree had a cluster of samples from the left and right sides of the primary tumor (LmidApicalCA, LapexCA, RmidApicalCA, and RapexCA), implying the widespread presence of some cancer cell populations. However, it is important to note that the tumor sample tree may be misleading in cases of multi-source seeding, and caution is warranted when interpreting its structure. Nevertheless, we found inferences from our example analysis were biologically plausible because tumor samples that were physically close to one another tended to cluster together, suggesting local tumor cell spread. Overall, GenoPath produces intuitive figures that assist users in analyzing metastatic tumor migration history and the relationship of tumor samples.

## Discussion

GenoPath integrates computational methods to analyze tumor evolution within a patient. By automating the execution of various evolutionary analyses and generating intuitive visual outputs, GenoPath reduces computational barriers, making advanced genomic research accessible to users with varying expertise levels. GenoPath is well-suited for analyzing data from various tumor DNA sequencing technologies, including whole-genome, whole-exome, and targeted sequencing.

We note that benchmarking of methods implemented in GenoPath has already been conducted in previous studies, where a wide range of tools were systematically evaluated (Miura et al., 2018; Miura et al., 2020; Miura et al., 2022; Kumar et al., 2020). GenoPath’s focus was to integrate these robust tools into a unified, user-friendly pipeline rather than re-evaluating their performance that is beyond the scope of this pipeline building. Importantly, insights from these previous studies also highlight key factors that influence the quality of tumor evolution inference. For example, larger numbers of somatic mutations and tumor samples enhance performance and improve the resolution of tumor evolution inference because of the limitations in clone prediction methods (Miura et al., 2020). Also, a larger number of mutations is essential for reliably identifying branch-specific mutational signatures, as observed mutational patterns for a branch of a clone phylogeny are often unclear from a smaller number of mutations, disabling identification of branch-specific mutational signatures (Miura et al., 2022).

While GenoPath currently implements selected computational methods, various alternative approaches exist (Roth et al., 2014; El-Kebir et al., 2015; Popic et al., 2015; Huzar et al., 2023). Future updates will incorporate additional well-performing methods, expanding its analytical capabilities. Additionally, GenoPath does not yet support the analysis of copy number alterations (CNAs) that also play a significant role in tumor evolution. Existing tools can infer clone phylogeny from CNAs or jointly analyze them with single-nucleotide variants (SNVs) (Jiang et al., 2016; Ricketts et al., 2020). Another current limitation is the lack of confidence estimates for the inferred phylogenies and migration paths. This is largely due to the fact that many of the existing methods do not natively provide statistical support metrics. Nonetheless, bootstrap-based approaches have been proposed in the literature (Huzar et al., 2023), and we plan to incorporate such methods into future releases to enhance the robustness of the evolutionary inferences provided by GenoPath. Lastly, GenoPath lacks partial run options, such as skipping migration inference. We also plan to add this flexibility in future updates.

Currently, GenoPath supports only bulk DNA sequencing data. However, with the increasing availability of tumor single-cell DNA sequencing data across various cancer types, there is growing interest in leveraging these datasets for evolutionary analysis (Navin et al., 2011; Borgsmüller et al., 2023). In fact, various computational methods for inferring cell phylogenies from single-cell sequencing are already available (Jahn et al., 2016; Kozlov et al., 2022; Miura et al., 2023). Importantly, once a cell phylogeny is constructed, the same downstream evolutionary analyses used for clone phylogenies can be applied. We plan to integrate computational methods for single-cell DNA sequencing data in future updates, expanding GenoPath’s functionality to accommodate bulk and single-cell analyses.

In conclusion, GenoPath offers practical advancements by integrating parsing and visualization modules that streamline the sequential execution of diverse software. These enhancements reduce the need for advanced computational expertise and make the interpretation of results more intuitive. By improving usability and accessibility, GenoPath serves as a versatile platform for tumor evolution analysis, facilitating discoveries across oncology, evolutionary biology, and precision medicine. We envision GenoPath as a broadly applicable resource that empowers biologists to gain deeper insights into cancer progression and heterogeneity.

## Data Availability

The original contributions presented in the study are included in the article/supplementary material, further inquiries can be directed to the corresponding author.
